# Advancing humanized 3D tumor modeling using an open access xeno-free medium

**DOI:** 10.3389/ftox.2025.1529360

**Published:** 2025-03-26

**Authors:** Atena Malakpour-Permlid, Manuel Marcos Rodriguez, Kinga Zór, Anja Boisen, Stina Oredsson

**Affiliations:** ^1^ Center for Intelligent Drug Delivery and Sensing Using Microcontainers and Nanomechanics (IDUN), Department of Health Technology, Technical University of Denmark, Lyngby, Denmark; ^2^ BioInnovation Institute Foundation, Copenhagen, Denmark; ^3^ Department of Biology, Lund University, Lund, Sweden

**Keywords:** HeLa cancer cells, MCF-7 cancer cells, cancer-associated fibroblasts (CAFs), 3D cell culture, high throughput drug screening, paclitaxel, Oredsson universal replacement (OUR) medium, FBS-free medium

## Abstract

Despite limitations like poor mimicry of the human cell microenvironment, contamination risks, and batch-to-batch variation, cell culture media with animal-derived components such as fetal bovine serum (FBS) have been used *in vitro* for decades. Moreover, a few reports have used animal-product-free media in advanced high throughput three-dimensional (3D) models that closely mimic *in vivo* conditions. To address these challenges, we combined a high throughput 3D model with an open access, FBS-free chemically-defined medium, Oredsson Universal Replacement (OUR) medium, to create a more realistic 3D *in vitro* drug screening system. To reach this goal, we report the gradual adaptation procedure of three cell lines: human HeLa cervical cancer cells, human MCF-7 breast cancer cells, and cancer-associated fibroblasts (CAFs) from FBS-supplemented medium to OUR medium, while closely monitoring cell attachment, proliferation, and morphology. Our data based on cell morphology studies with phase contrast and real-time live imaging demonstrates a successful adaptation of cells to proliferate in OUR medium showing sustained growth kinetics and maintaining population doubling time. The morphological analysis demonstrates that HeLa and MCF-7 cells displayed altered cell morphology, with a more spread-out cytoplasm and significantly lower circularity index, while CAFs remained unaffected when grown in OUR medium. 3D fiber scaffolds facilitated efficient cell distribution and ingrowth when grown in OUR medium, where cells expand and infiltrate into the depths of 3D scaffolds. Drug toxicity evaluation of the widely used anti-cancer drug paclitaxel (PTX) revealed that cells grown in 3D cultures with OUR medium showed significantly lower sensitivity to PTX, which was consistent with the FBS-supplemented medium. We believe this study opens the way and encourages the scientific community to use animal product-free cell culture medium formulations for research and toxicity testing.

## Graphical Abstract

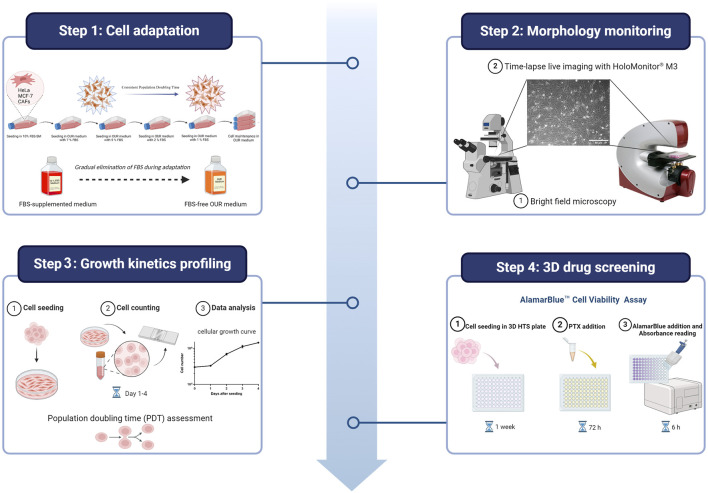

## 1 Introduction

Conventional two-dimensional cell cultures (2D) provide an artificial set-up for culturing cells in a monolayer that significantly differs from the growth condition of cells within tumors due to the lack of structural and biochemical composition found in human tumors *in vivo* (Jensen and Teng, 2020; Ackermann and Tardito, 2019). Recently, 3D cell culturing approaches based on patient-derived organoids, spheroids, and other scaffold-based 3D systems have emerged to enhance the accuracy and efficacy of cancer research and drug screening (Cacciamali et al., 2022). Scaffold-based 3D tumor models can provide both spatial and structural support and biophysical signals to the cells, essential for mimicking the tumor architecture, morphogenesis, and tumor-stromal crosstalk (Barbosa et al., 2022). Despite these advances, most of the ongoing cancer research is still performed using conventional cell growth media supplemented with fetal bovine serum (FBS) formulated over half a century ago (Ackermann and Tardito, 2019). FBS is a source to easily obtain growth factors, essential hormones, minerals, and trace elements required for attachment and proliferation for long-term cell culturing and use of cells in different experimental settings. Regardless of the ethical issues concerning FBS production and animal welfare (Van der Valk et al., 2018), FBS has a high variability in the concentration of unknown and ill-defined components, which could lead to significant batch-to-batch variation which may affect scientific reproducibility (Oredsson et al., 2019; Jochems et al., 2002). Moreover, FBS is a recognized source of viral and prion (xenogeneic proteins) contamination in cell culture due to the risk of bovine diseases and non-human pathogens transmission (Pilgrim et al., 2022; van der Valk, 2022; Weber et al., 2022). Furthermore, certain FBS batches may contain toxic factors that can significantly affect the quality and reliability of the experimental results (van der Valk, 2022).

To address the issues regarding the use of FBS in cell culture media there are several commercially available animal-free components media alternatives in the market (*e.g.*, animal origin component-free Cytiva Life Sciences HyClone™ serum-free medium, and Gibco Dynamis™ serum-free medium for growing mammalian cells) as well as medium supplemented with human serum or human platelet lysate (Chelladurai et al., 2021). These xeno-free media alternatives aim to replace FBS supplementation in cell culture (Pilgrim et al., 2022; Caneparo et al., 2022). However, many of these animal-component-free media lack open access for commercial and proprietary reasons and the specific media formulations are often undisclosed, making scientific progress in the field more challenging (van der Valk, 2022). Therefore, among the research community, many research groups have attempted to formulate a serum-free medium to overcome these limitations. Establishing open access sources, and databases such as https://fcs-free.org/ and encouraging information-sharing on the formulation details of serum-free medium are crucial for advancing research practices in the field. Serum-free medium allows scientific consistency and reproducibility across different laboratories and research experiments while alleviating the issues related to variability, contamination, and ethics (Gottipamula et al., 2013). To this end, we have successfully developed an open source, animal component-free, and chemically defined medium (Rafnsdóttir et al., 2023; Weber et al., 2022). This defined medium, which we now refer to as the Oredsson Universal Replacement (OUR) medium (Oredsson et al., 2024), is formulated to support a wide variety of cell types, both adherent and suspension cells, and offers a step forward in xeno-free 3D tumor modeling. Serum-free media has been frequently used to obtain patient-derived tumor organoids and stem-cell-derived organoids (Balvers et al., 2013; Pamarthy and Sabaawy, 2021). This approach was initiated by the Sasai group in 2008, which used the serum-free medium for generating self-organized brain organoids (Eiraku et al., 2008). However, a few studies have demonstrated the use of serum-free media in scaffold-based 3D culture systems in cancer research. Gomez-Roman et al. (2017) developed a 3D glioblastoma model using Alvetex polystyrene scaffold in combination with a serum-free medium to study anti-cancer drug and radiation responses. This suggests that further optimization of the dual use of serum-free media with scaffold-based 3D systems is needed to develop clinically relevant tumor models.

In the present study, we aim to recreate a physiologically relevant 3D human cervical and breast mini-tumor model in combination with xeno-free OUR medium within a high throughput screening (HTS) system. The 3D cultures provide a more physiologically relevant context and by combining this with OUR medium, this approach represents a better and more promising alternative model for *in vitro* drug testing and investigation of tumor biology. The human cancer cell lines and cancer-associated fibroblasts (CAFs) used in this study have been gradually adapted and transitioned from a conventional 10% FBS-supplemented medium to OUR medium. Throughout the adaptation procedure, several key cell parameters such as proliferation rate and morphology were closely monitored. We employed biocompatible, collagen-mimicking electrospun polycaprolactone (PCL)-based 3D 96-well plates developed and validated previously by Malakpour-Permlid and Oredsson (2021). Here, we use the 3D scaffolds to generate 3D mono-cultures of human cervical cancer HeLa cells, human breast cancer MCF-7 cells, and CAFs in an animal-component-free setup. The cytotoxicity of the conventional chemotherapeutic drug, paclitaxel (PTX) was assessed in 3D HTS cultures. We propose that our xeno-free 3D tumor model setup serves as an alternative to standard 3D drug screening platforms that typically rely on the use of FBS-supplemented medium and other animal-derived components.

## 2 Materials and methods

### 2.1 Cell lines

The human breast cancer cell line MCF-7 (HTB-22) and the human cervical carcinoma cell line HeLa (CCL-2) were purchased from the American Type Culture Collection (Manassas, VA, United States). Human CAFs were obtained from Prof. Stina Oredsson, Lund University with permission to use from Prof. Akira Orimo, Graduate School Juntendo University, Tokyo, Japan (Kojima et al., 2010). Before adaptation to OUR medium, all the cell lines were cultured in Dulbecco’s Modified Eagle Medium containing 4.5 g/L glucose (Sigma-Aldrich, Denmark A/S, Hellerup, Denmark) supplemented with 10% heat-inactivated FBS (Sigma-Aldrich, Denmark A/S, Hellerup, Denmark), 2 mM L-glutamine (ThermoFisher Scientific, Waltham, MA, United States), 1 mM non-essential amino acids (ThermoFisher Scientific), 100 μg/mL streptomycin (Gibco, Gaithersburg, MD, United States), and 100 U/mL penicillin (Gibco). The cell lines were passaged twice a week using trypsin/EDTA™ (0.05%) (Sigma-Aldrich Sweden AB) while they were maintained in a humidified incubator (95% humidity) with 5% CO_2_ in air at 37°C in CO_2_ incubator.

### 2.2 Drug treatment

The conventional chemotherapeutic drug PTX was obtained from Tocris Bioscience (Abingdon, United Kingdom). A 100 mM stock solution was made in 100% dimethyl sulfoxide (DMSO) (Sigma-Aldrich, Denmark A/S, Hellerup, Denmark) and stored at −20 ^°^C. A working solution of 100 µM was diluted in sterile PBS with no calcium or magnesium (Gibco). A serial dilution using the medium with 1,000, 500, 250, 100, 50, and 10 nM final concentrations was prepared and used for the experiments. The cells treated with PBS with 0.1% DMSO were considered as standard negative control. Additionally, as positive controls, DMSO was used to create a serial dilution with concentrations of 0.1, 0.6, 1.2, 2.5, 5, 8, and 10% in OUR medium.

### 2.3 Cell adaptation to OUR medium

**FIGURE 1:**
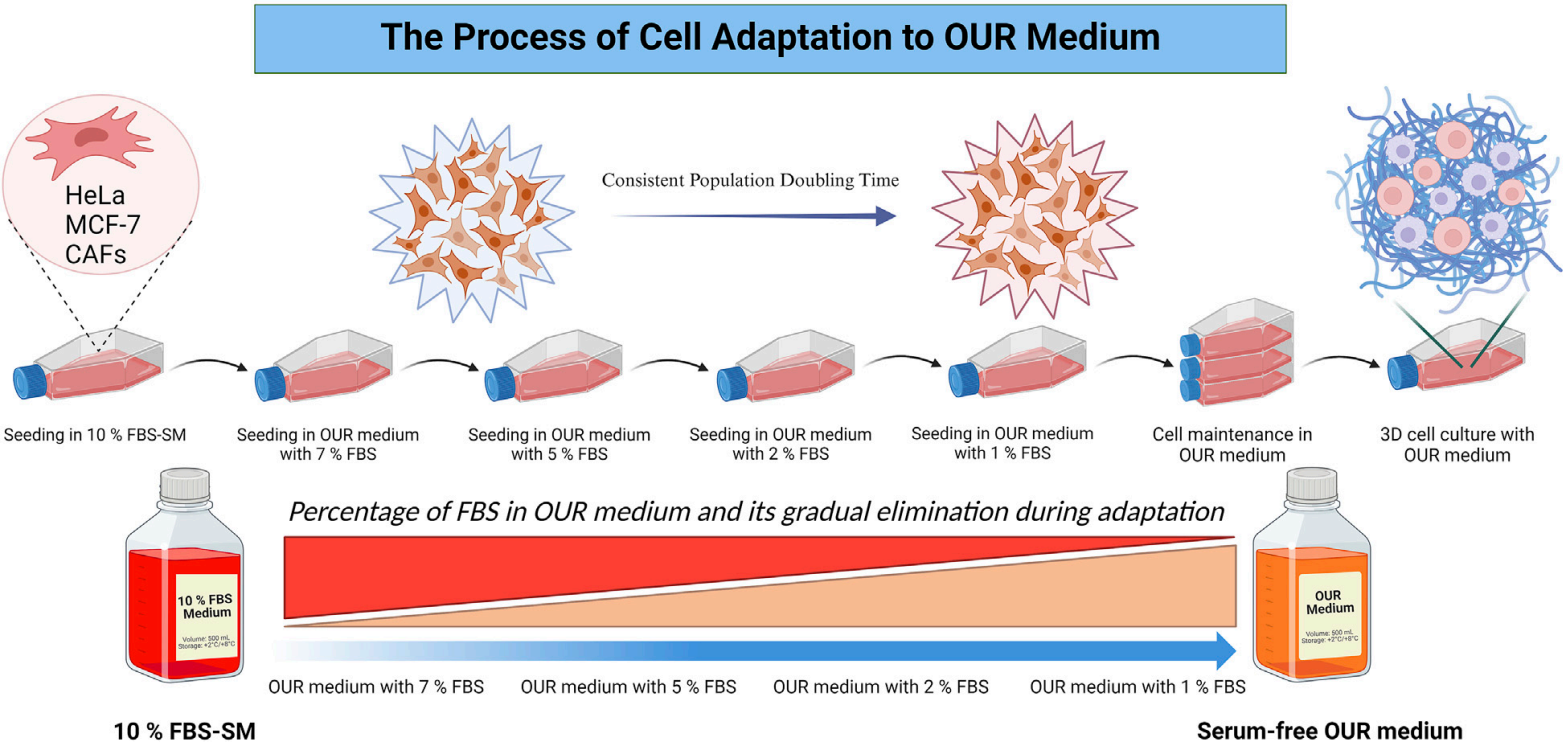
Schematic illustration of the process involved in cell adaptation from an FBS-supplemented medium (FBS-SM) to Oredsson Universal Replacement (OUR) medium.

The composition and formulation of OUR medium used in this study were previously described and specified by Rafnsdóttir et al. (2023) and Weber et al. (2022). The OUR medium is an animal product-free, chemically defined medium designed for both 2D and 3D cell culture. As described by Rafnsdóttir et al. (2023), it contains DMEM/Ham´s F12 as a basal medium with a mix of non-protein and protein components essential for cell attachment, spreading, proliferation, and long-term routine cell culturing. To promote optimal cell attachment and proliferation in OUR medium, all the cell lines underwent a 1-month adaptation period where they were introduced and adapted to OUR medium by gradual dilution of FBS as illustrated in Figure 1. Initially, all the cells were cultured in a DMEM medium supplemented with 10% FBS. Then, the reduction procedure was performed in a step-wise manner, gradually decreasing the FBS content in OUR medium (7%, 5%, 2%, 1%, and 0%), ultimately leading to the complete elimination of FBS in OUR medium. Before introducing the cells to the reduced percentages of FBS in OUR medium the cells were passaged and pelleted by centrifugation at 300g for 5 min at 4°C. Then, OUR medium was removed, and the cell pellet was resuspended in the fresh OUR medium with the lower percentages of FBS until FBS was totally omitted. To improve cell proliferation, attachment, and expansion, Corning^®^ Primaria cell culture flasks (Corning, New York, United States) were used instead of standard cell culture flasks during the adaptation and routine cell culturing. The light in the laminar flow bench was turned off due to the medium’s sensitivity to light, due to the potential degradation of certain components when exposed to light. The cells were defined as adapted when the cell growth between routine passaging was constant showing stable cell growth characteristics, such as a consistent doubling time and stable morphology.

### 2.4 Cell culture maintenance

The cells grown in OUR medium were initially rinsed with PBS when passaging. Following aspiration of PBS, the animal-free recombinant trypsin-like enzyme (Tsuji et al., 2017), TrypLE™ Select dissociation reagent (ThermoFisher Scientific) was used (1.5 mL/75 cm^2^) for detachment and the cells were incubated at 37°C for 5 min. Subsequently, 5 mL of OUR medium was added, and the cells were dispersed through careful trituration. Another 5 mL of OUR medium was then added, and the cell numbers were determined using an automated cell counting unit (NucleoCounter^®^ NC-200TM, ChemoMetec A/S, Allerod, Denmark). After cell counting, 3 × 10^6^ cells were transferred to a 75 cm Corning^®^ Primaria cell culture flask. The cells were passaged twice a week with fresh medium replacement after 72 h in between sub-culturing. The replacement medium did not contain fibronectin (Rafnsdóttir et al., 2023). Routine passaging and the following experimental work were carried out with the light turned off in the laminar flow bench due to the light sensitivity of the components of the OUR medium. The cells were maintained in a humidified incubator (95% humidity) with 5% CO_2_ in air at 37°C (CO_2_ incubator).

### 2.5 High throughput 3D polycaprolactone-based 96-well plates

For the drug screening experiments, we used a custom-designed high throughput (HT) non-homogenous 3D PCL fiber network incorporated into the wells of 96-well plate as described previously by Malakpour-Permlid and Oredsson (2021). In short, to obtain a non-homogenous PCL fiber structure, the electrospun PCL fiber membranes were sectioned into well-defined fragments within a semi-solid ethanol/water mixture (4 mg/mL) using a sterilized Waring LB20ES laboratory mixer (Radnor, PA, United States) for 15 min. To maintain a temperature below −60°C, which is the glass transition point of PCL, liquid nitrogen was periodically added to the PCL mixture at a rate of 10 mL per 100 mL of mixture. Then, PCL solution (150 μL) was dispensed into the wells of clear flat-bottomed hydrophobic 96-well plates (Corning, NY, United States). The 96-well plate with PCL solution in the wells was incubated at 59°C for 7 min, followed by immediate placement on ice. Lastly, the plates were left to dry overnight in a laminar airflow bench.

### 2.6 Surface modification by plasma treatment and sterilization

As reported by Recek et al. (2016) and Asadian et al. (2020), to enhance the hydrophilicity of PCL fibers in the wells of 96-well plates, the plates underwent a 10 s O_2_ plasma treatment using a ZEPTO low-pressure plasma laboratory unit (Diener electronic GmbH Co. Ebhausen, Germany). Before seeding cells into the 3D 96-well plates, they were sterilized with absolute ethanol for 15 min, followed by rinsing the wells thrice with sterile PBS.

### 2.7 Growth curve studies

HeLa cells, MCF-7 cells, and CAFs used for growth curve experiments were seeded in Primaria Petri dishes with a diameter of 3 cm. All cell lines were seeded at a density of 32,000 cells/cm^2^. On days 1, 2, 3, and 4 after seeding, cells in Petri dishes were dislodged using TrypLE™ Select dissociation reagent (500 µL) and resuspended in fresh OUR medium before cell counting with NucleoCounter^®^ NC-200TM automated cell counting unit (ChemoMetec A/S, Allerod, Denmark). All the growth curve experiments were repeated 3 times, each time with 2 replicates (n = 6). The population doubling time (PDT) was calculated using the following formula:

$$PDT=\frac{T\;x\;\log\left(2\right)}{\log\left(Nt\right)-\log\left(N0\right)}$$

Where T is the duration of the growth period, Nt represents the final cell count at time t, N0 is the initial cell count at time 0, and t represents the time interval between the initial and final cell counts.

### 2.8 Cell morphology analysis

Cell morphology was observed and visualized using a Zeiss bright-field primovert inverted phase contrast microscope (Carl Zeiss Suzhou Co., Ltd., Suzhou, China) using 10 × 0.3 NA. objective lenses. The average cell area and circularity index were determined to quantify the cell morphology parameters. Data were collected from 60 different cell measurements for each cell type derived from 3 different phase contrast images. ImageJ (www.imagej.net/) was used for subsequent cell area and circularity index analysis using the following formula:

$$circularity\;index=4\pi \;\left(area/{perimeter}^{2}\right)$$

According to the ImageJ program, the circularity index value of 1.0 indicates a perfect circle while a value approaching 0.0 suggests an increasingly elongated or polygonal morphology (https://imagej.net/ij/plugins/circularity.html).

### 2.9 Cell seeding in 3D cultures for drug screening

After cell detachment, the cells were resuspended in OUR medium, and cell suspensions containing a defined number of HeLa cells, MCF-7 cells, or CAFs were prepared using OUR medium (500,000 cells/mL), and 100 µL of cell suspension (50,000 cells) was added carefully on top of 3D scaffolds in the wells of 3D 96-well plates. The cells were allowed to adhere to the network of the PCL-based 96-well plates for 2 h followed by the addition of 100 µL medium. The plates were kept in the CO_2_ incubator for 1 week and with OUR medium renewal after 72 h of incubation. After 1 week of incubation, the medium was changed, and the final concentrations shown in figures of PTX or DMSO in OUR medium were added to the wells. The chosen concentrations used in these experiments are based on the work of Zasadil et al. (2014), Malakpour-Permlid and Oredsson (2021), and Rafnsdóttir et al. (2023) defining clinically relevant doses of paclitaxel for drug screening. Next, the cultures were incubated in the CO_2_ incubator for 72 h.

### 2.10 AlamarBlue™ cell viability assay

Cell viability was indirectly assessed by measuring the reduction of AlamarBlue™, a colorimetric indicator of cellular mitochondrial activity (O'Brien et al., 2000). The AlamarBlue™ reagent (ThermoFisher Scientific) was added (20 µL) to the medium of each well of the plate. The plate was covered with aluminum foil to avoid exposure to light and incubated in the CO_2_ incubator for 6 h. Then, 100 µL of the medium in each well was transferred to another standard 96-well plate to measure the reduced resazurin to resorufin in the medium. The measurement was performed using a Tecan Spark 20 M microplate reader, operated with Tecan Spark Control dashboard software (Tecan, Männedorf, Switzerland). The reduction of AlamarBlue™ was measured at 590 nm emission after excitation at 540 nm. The results are plotted as % of control (Figure 6) *i.e.*, fluorescence units at 590 nm. All the experiments were repeated at least three or four times.

### 2.11 Fixation and staining of cells for visualization

Following the AlamarBlue™ assay, the cell cultures were fixed with 3.7% formaldehyde in PBS and maintained at 4°C for 15 min. After this, the cells were washed with PBS three times. Blocking and permeabilization of the cultures were achieved by adding a blocking buffer with 1% bovine serum albumin and 1% Tween 20 in PBS at room temperature for 1 h. Then, the cultures were incubated with Alexa Fluor™ 488 phalloidin (A11029, 1:50) (ThermoFisher Scientific) for 2 h at room temperature. Following three washes with PBS, the cell nuclei were stained with 4′,6-diamidino-2-phenylindole (DAPI) at 1 μg/mL concentration in PBS for 2 min at room temperature. The stained cultures were stored at 4°C overnight before microscopic imaging. We currently use human serum albumin to replace bovine serum albumin during the staining procedure.

### 2.12 Confocal laser scanning microscopy

To examine the distribution of the cells in the 3D PCL network, a Leica SP8 DLS inverted confocal laser scanning microscope (Leica Microsystems, Wetzlar, Germany) was used. Imaging was performed using a 25×/0.95 IMM water immersion objective. For 3D cultured samples, RapiClear^®^ 1.49 clearing agent from SunJin Lab Co. (Hsinchu, Taiwan) was applied to facilitate imaging. Each culture condition was imaged in at least three replicates, with 3–5 images captured per replicate. This ensured that a representative sample of the cells was captured, and reliable data was obtained for analysis.

### 2.13 Time-lapse live microscopy

For time-lapse live imaging of cell proliferation and movement, we used a HoloMonitor^®^ M3 phase holographic microscope (PHI AB, Lund, Sweden) which was placed in a regular 37°C CO_2_ incubator. The cells were seeded in 25 cm^2^ Primaria tissue culture flasks and they were allowed to attach for 24 h in a 37°C CO_2_ incubator before initiating the time-lapse imaging. The vented cap was tightly screwed and then the tissue culture flask was placed on the stage of the HoloMonitor^®^ M3 phase holographic microscope, and images were captured every 10 min for 72 h with a ×10 phase contrast objective. For image acquisition on the M3 microscope, the software program Hstudio^®^ (PHI AB) was used.

### 2.14 Statistical analysis

The experiments conducted in this study were repeated at least three or four times, with five to six independent cultures utilized in each experimental iteration. The results obtained were expressed as the mean ± standard error of the mean (SEM). When two groups were compared, Student’s t-test was used and a p-value less than 0.05 (p < 0.05) was considered statistically significant. GraphPad Prism 10 software (www.graphpad.com) was used to plot the graphs. The graphical abstract and illustration 1 were created using the BioRender platform (www.BioRender.com).

## 3 Results

### 3.1 Cell morphology and microscopic appearance after adaption to OUR medium

When adapting immortalized cell lines to a serum-free medium, typically two different fast and slow adaptation approaches are followed (van der Valk et al., 2010). Either a rapid adaptation can be employed in which the cells are directly adapted to the serum-free medium by a direct and abrupt switch from FBS-supplemented medium to serum-free medium (Sinacore, et al., 2000) or a step-wise adaptation procedure where it involves gradual reduction of FBS-concentrations over multiple passages (van der Valk et al., 2010; Weber et al., 2022). Here, we decided to use a gradual reduction adaptation protocol to OUR medium following Rafnsdóttir et al. (2023) where the FBS was serially diluted to finally reach 0%. The cells were initially cultured in a 10% FBS-supplemented medium and the FBS concentration was gradually reduced during each subsequent passaging from 10% to 7%, to 5%, to 2%, to 1%, and 0% in OUR medium until the cells could be grown completely serum-free OUR medium. Also, cell characteristics such as attachment, morphology, and proliferation were closely monitored during each adaptation step in this work.

**FIGURE 2:**
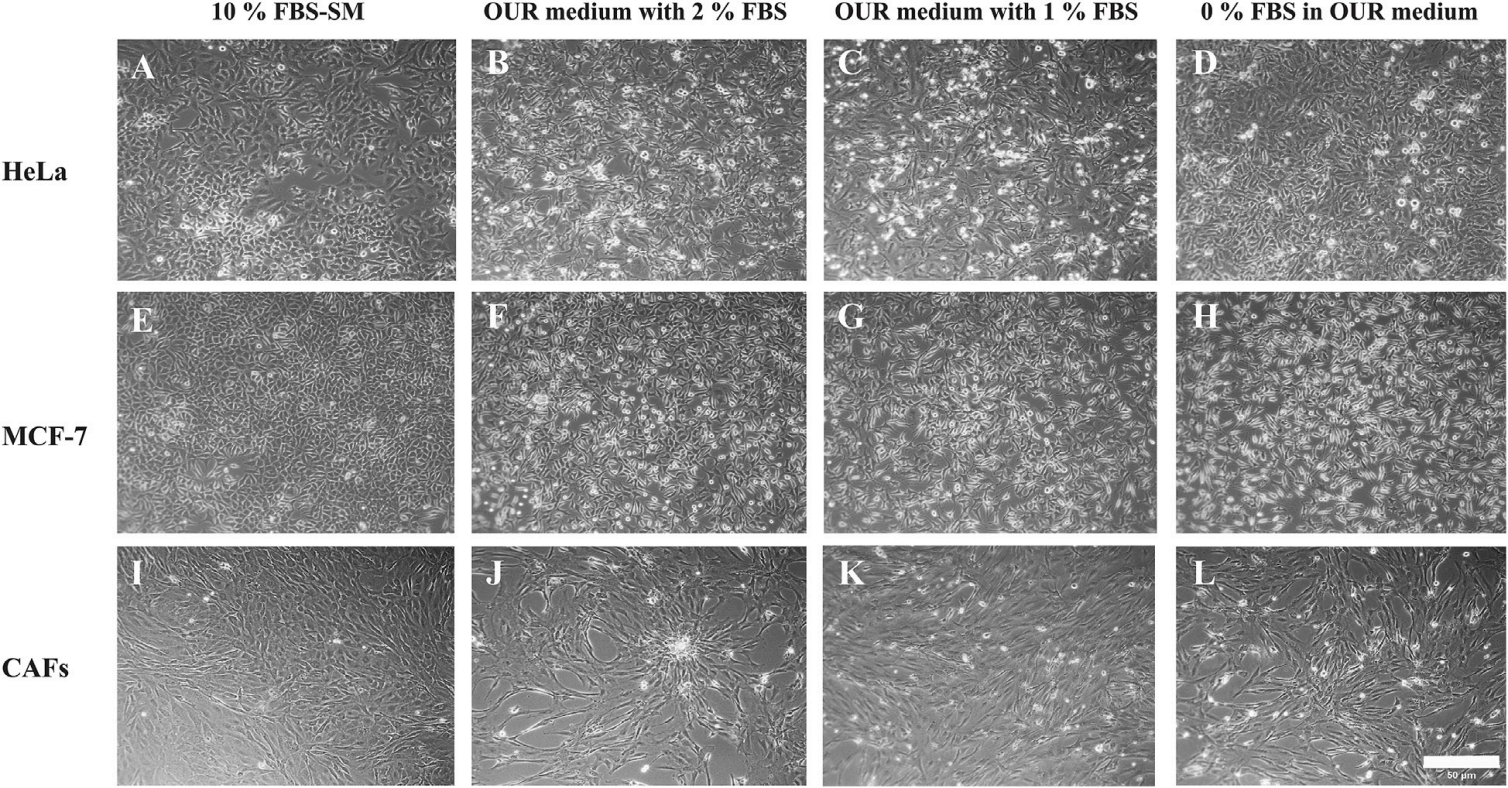
Morphology of human cervical cancer HeLa cells **(A–D)**, human breast cancer MCF-7 cells **(E–H)**, and cancer-associated fibroblasts (CAFs) **(I–L)** cultured in medium with 10% FBS or in OUR medium supplemented with 2, 1, or 0% FBS, respectively. Representative images have been taken with an inverted phase contrast microscope at ×10 magnification. The scale bar is 50 µm.

**FIGURE 3:**
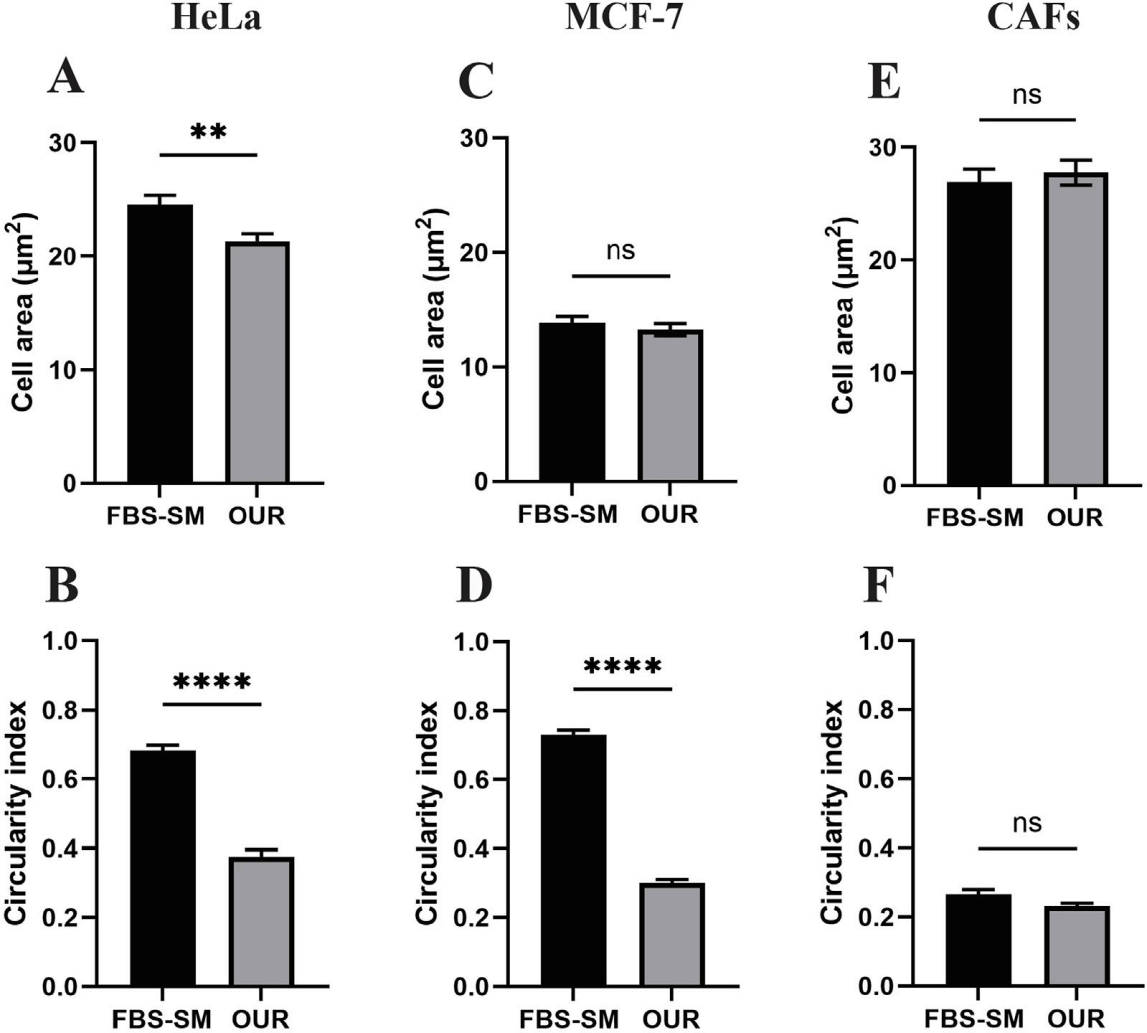
Morphological analysis of cell area and circularity index of human cervical cancer HeLa cells **(A, B)**, human breast cancer MCF-7 cells **(C, D)**, and cancer-associated fibroblasts (CAFs) **(E, F)** cultured in medium supplemented with 10% FBS (FBS-SM) and OUR medium, respectively. The cell area and circularity index were analyzed using ImageJ software. Results represented mean ± SEM of three independent images (N = 3, n = 60).

All the cell lines adhered to the Corning^®^ Primaria tissue culture vessels after passaging in each adaptation step. We have utilized the Primaria cell culture flasks instead of conventional or regular plastic tissue culture vessels recommended by Ince et al. (2007) in this work. The cells were allowed to grow and proliferate for a week to reach approximately 90% confluency before the next passaging. Each cell line was then cultured and maintained in the same reduced FBS-supplemented medium for 2–3 subculture before proceeding with the following serum reduction steps. All the cell lines eventually were successfully adapted to the OUR medium. To compare the effect of different growth culture media on the phenotypic behavior of cells, the morphology of the MCF-7 cells, HeLa cells, and CAFs were evaluated using microscopic imaging during the adaptation process to ensure the establishment of a stable and well-adapted cell line. Representative images of each cell type in a medium with 10% FBS and then adapted to OUR medium containing 2% FBS, 1%, and FBS-free OUR medium are shown in Figure 2. Further, changes in the morphology of cells cultured in 10% FBS-supplemented medium and OUR medium after complete adaptation were evaluated by analyzing the cell area and circularity index, as shown in Figure 3. The cells did not show any difference in morphology during the initial adaptation process with 7% FBS, and 5% FBS in OUR medium. However, when cultured in OUR medium without FBS, HeLa (Figure 2D) and MCF-7 (Figure 2H) cancer cells exhibit a more spread-out morphology with extended cytoplasm compared to their appearance when grown in FBS-supplemented, where they tended to have a more circular and polygonal morphology. This observation aligns with our cell circularity index analysis, showing that HeLa and MCF-7 cells grown in OUR medium demonstrate significant elongation, with their circularity index being significantly lower compared to when grown in an FBS-supplemented medium (Figures 3B, D). However, it was interesting to observe that while OUR medium significantly reduced the area of HeLa cells, MCF-7 maintained its size regardless of the elongation effect (Figures 3A, C). In contrast, no significant difference was observed in the morphology of CAFs when grown in FBS-supplemented and OUR medium (Figures 2I, L). This result is consistent with CAFs cell area and circularity index analysis when grown in both media (Figures 3E, F).

### 3.2 Live real-time observation of cell proliferation and movement

In addition to monitoring cell phenotype at specific time points, we have observed HeLa cells, MCF-7 cancer cells, and CAFs behavior in real-time for 72 h using time-lapse imaging in an M3 holographic microscope. Supplemental videos S1–S3 are M3-derived phase contrast time-lapse videos of HeLa, MCF-7, and CAFs, respectively, cultured in OUR medium for 72 h. In the movies, cells are seen rounding up and dividing into 2 cells, after which they spread out and attach to the plates. The gradual increase in cell numbers is obvious as well in cell movement. Supplemental videos S1 shows HeLa cells exhibiting rapid movements with cytoplasmic protrusions, indicating active cell motility and dynamic changes in cell morphology during proliferation and cell division. Supplemental video S2 shows MCF-7 cells displaying similar proliferative behavior, with many cell divisions leading to a steady increase in cell numbers and growth patterns throughout the imaging period. In Supplemental videos S3, CAFs are observed to increase in cell number progressively showing a distinctive elongated and thin morphology. The time-lapse movie highlighted their tight interactions and frequent burst divisions. Unlike HeLa and MCF-7 cells, CAFs move around together more collectively while extending and retracting their cytoplasm during cell division. Overall, the time-lapse videos comprehensively show the dynamic cellular activities, behaviors, and proliferation of HeLa, MCF-7, and CAF cells during routine culturing in OUR medium.

### 3.3 Cell proliferation and growth after adaption to OUR medium

**FIGURE 4:**
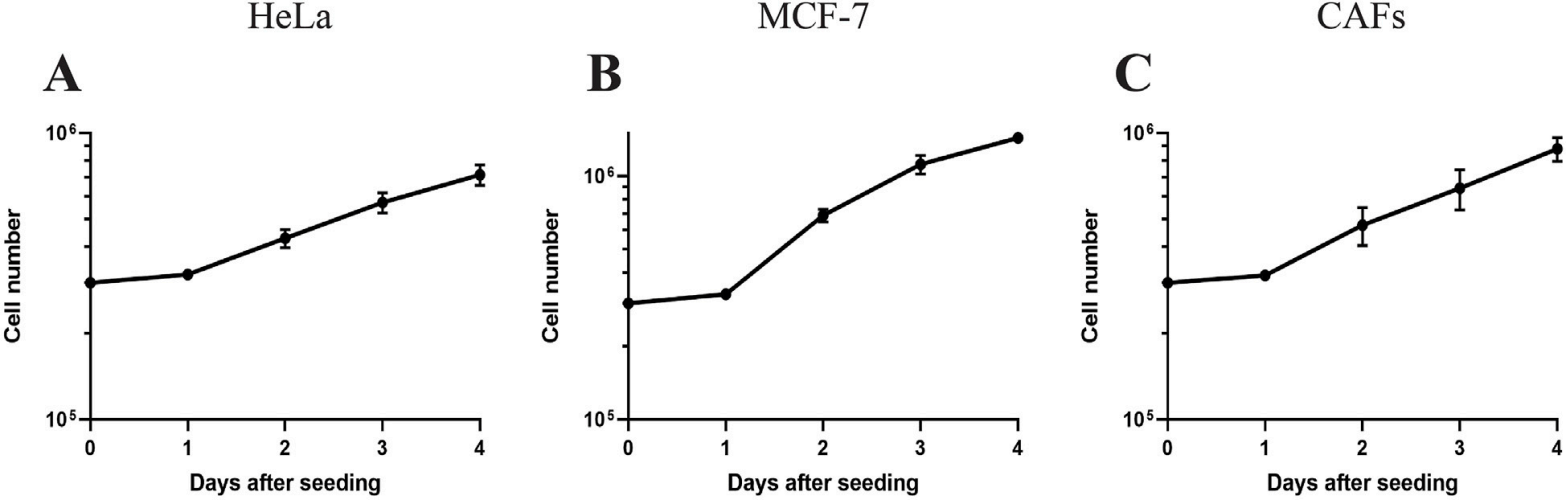
Growth curves of human cervical cancer HeLa cells **(A)**, human breast cancer MCF‐7 cells **(B)**, and cancer-associated fibroblasts (CAFs) **(C)** cultured in OUR medium in a 2D monolayer system. The data for each cell line is compiled from 3 independent experiments (where the number of replicates in each experiment is 3 or 4). The symbol represents the mean of the cell number of all data ± SEM.

Following the FBS reduction step, we consistently maintained the cells in several routine passages, during which optimal growth was established. Optimal growth was indicated when the cells showed consistent cell proliferation, stable cell numbers at every routine passage, and reached confluency over several days. Consequently, the cells were considered to have successfully adapted to OUR medium and were ready for further experiments. As the components of the growth medium can impact cell performance and behavior (Caneparo et al., 2022), in the next step, we aimed to establish growth curves of HeLa and MCF-7 cancer cells and CAFs to determine their PDTs (Figure 4). The growth kinetics of mammalian cell subculturing commonly consists of an initial lag phase where the cells adapt to their new environment. This phase is followed by a phase of exponential growth where the PDT can be calculated (Sakthiselvan, et al., 2019). All the cell lines grown in OUR medium show a 24 h lag phase before entering the exponential phase of cell proliferation (Figure 4). Table 1 presents the PDTs of HeLa and MCF-7 cancer cells as well as of CAFs cultured in OUR medium and 10% FBS-supplemented medium. The data are derived from our laboratory and obtained from the literature for comparison. Additionally, we have deduced the PDT of all the cells cultured in FBS-supplemented medium from routine passaging during this study. The PDT during the exponential growth phase was 57.5, 32, and 46.3 h for HeLa cells, MCF-7 cells, and CAFs in OUR medium, respectively (Table 1). MCF-7 and CAFs proliferated at a growth rate in OUR medium comparable to that in the FBS-supplemented medium, but not HeLa cells. When culturing HeLa cells in OUR medium, HeLa cells proliferate slower compared to the proliferation in the FBS-supplemented medium. According to the Cellosaurus cell lines database, PDT of HeLa cells grown in an FBS-supplemented medium is reported to be 31.2 h and 48 h from two different sources (www.cellosaurus.org/CVCL_0030). The PDT of MCF-7 cells and CAFs cultured in the OUR medium were comparable to those for the same cell lines cultured in FBS-supplemented in our laboratory and as found in the literature (Table 1). Cellosaurus cell lines database has reported a PDT of 31.2 h for MCF-7 cells in an FBS-supplemented medium (www.cellosaurus.org/CVCL_0031). Additionally, the National Cancer Institute (NCI) has reported a doubling time of 25.4 h for MCF-7 cells (NCI-60 cancer cell line list: https://dtp.cancer.gov/discovery_development/nci-60/cell_list.htm). PDT of CAFs cultured in an FBS-supplemented medium in our laboratory was found to range between 38–45 h depending on the cell’s passage number and it is known that doubling times for CAFs can vary significantly due to their extreme heterogeneity (Louault et al., 2020; Ored et al., 2020).

**TABLE 1:** Population doubling time (PDT) of human cervical cancer HeLa cells, human breast cancer MCF-7 cells, and cancer-associated fibroblast (CAFs) cultured in OUR medium and in 10% FBS-supplemented medium.

Cell line medium type		References	PDT (hours)
HeLa	OUR medium	Present data	57.5
	10% FBS-supplemented medium	Routine culturing^a^	22–32
	10% FBS-supplemented medium	Tang (2019)	17.5–32.3
MCF-7	OUR medium	Present data	32
	10% FBS-supplemented medium	Routine culturing^a^	34
	10% FBS-supplemented medium	Larsson et al., 2008; Silva et al., 2015	34, 35
CAFs	OUR medium	Present data	46.3
	10% FBS-supplemented medium	Routine culturing^a^	38–45
	10% FBS-supplemented medium	Liu et al. (2006)	48–60

^a^ The PDT, is obtained from cell counting during routine cell culturing in this study.

### 3.4 Evaluation of cell expansion and infiltration into 3D cultures using OUR medium

**FIGURE 5:**
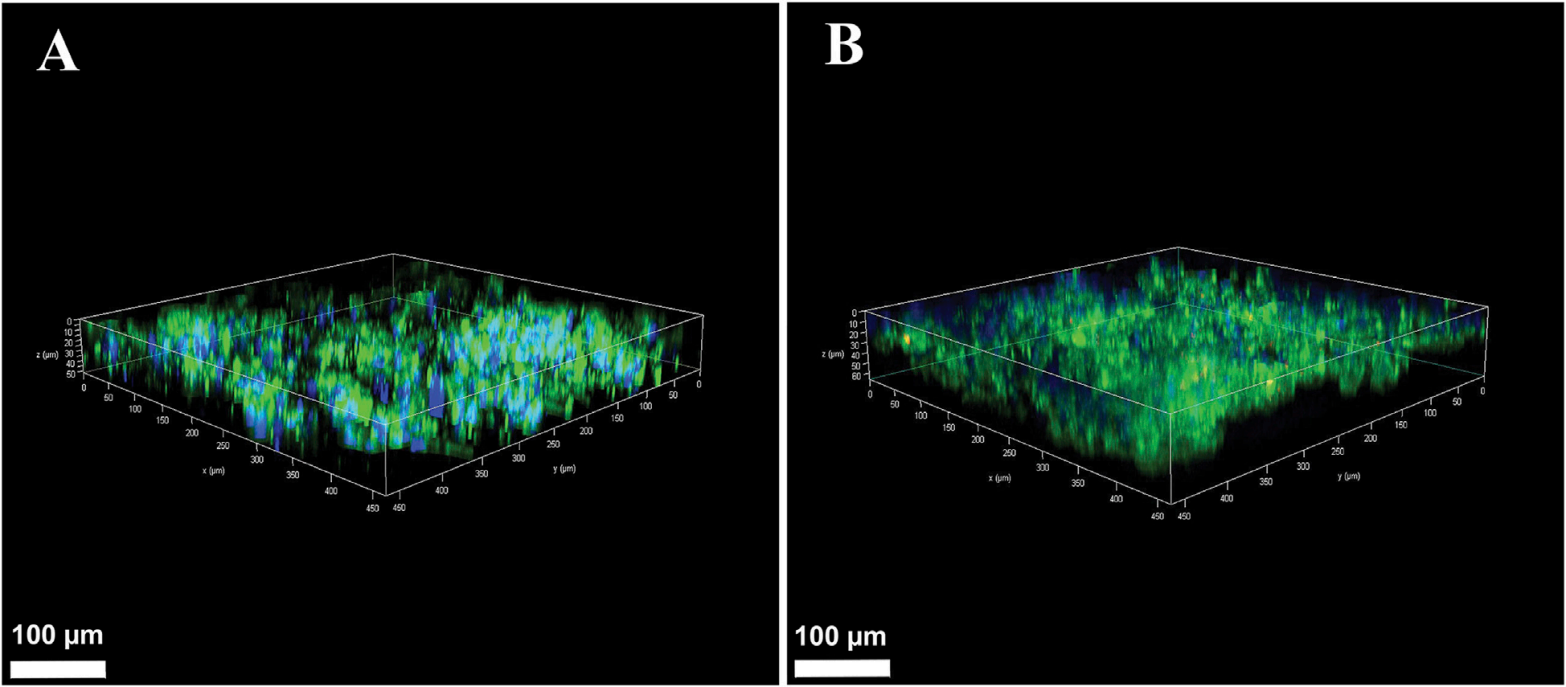
Confocal microscopy Z-stack images of human cervical cancer HeLa cells **(A)** and human breast cancer MCF-7 cells **(B)** in 3D cultures after 10 days of incubation in OUR medium. The cultures were fixed in 4% formaldehyde and labeled to visualize actin filaments (green) and cell nuclei (blue). The confocal plane images were obtained at approximately 2 µm distance from each other in the 3D culture. The scale bars are 100 µm.

After establishing the cell proliferation rate in OUR medium, we aimed to grow cells in an HTS 3D cell culturing model to evaluate the suitability of OUR medium to support the expansion and infiltration of cells into a PCL-based fiber network scaffold. HeLa and MCF-7 cells were cultured in a 3D PCL-based fiber network in OUR medium for 10 days to investigate cell expansion and infiltration. Confocal z-stack imaging was performed to assess the infiltration of HeLa and MCF-7 cells grown in OUR medium into the 3D culture model (Figure 5). The results from the images show that cells cultured in OUR medium were able to infiltrate into the depth of the 3D PCL fiber network (Figure 5). Our z-stack imaging data show infiltration to a depth of 50 μm and 62 µm for HeLa and MCF-7 cells, respectively (Figures 5A, B). These findings demonstrate, for the first time, that OUR medium successfully supports the infiltration of both HeLa and MCF-7 cells into the depth of the 3D PCL fiber network.

### 3.5 Drug toxicity of cells in 3D cultures grown in OUR medium

**FIGURE 6:**
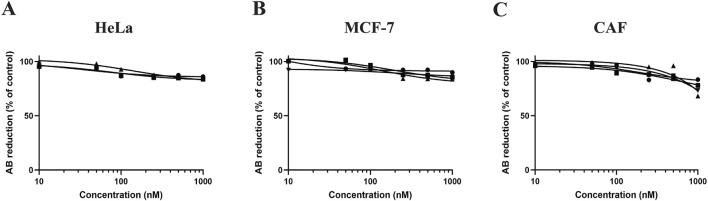
Dose-response curves for human cervical cancer HeLa cells **(A)**, human breast cancer MCF cells **(B)**, and cancer-associated fibroblasts (CAFs) **(C)** in 3D cultures treated with paclitaxel (PTX). The cells were seeded and incubated for 1 week in 3D cultures in OUR medium before adding PTX at concentrations 10, 50, 100, 250, 500, and 1,000 nM. Control cells were treated with PBS containing 0.1% DMSO. The toxicity was evaluated using the AlamarBlue™ assay and the results are expressed as the percentage reduction of AlamarBlue™ compared to control. The curves are drawn in GraphPad Prism 10 using all data from three or four independent experiments with n = 18–24 for each data point in the figure. The triangle, circle, and square symbols represent different experimental replicates.

In this study, we investigated the toxicity of PTX on HeLa, MCF-7 cells, and CAFs cultured in OUR medium in 3D HTS 96-well plates. Figure 6 displays the dose-response curves derived from three or four independent experiments in which HeLa, MCF-7, and CAF cultures were treated with PTX for 72 h (Figures 6A–C). Control or untreated cells were exposed to PBS containing 0.1% DMSO for 72 h. The results show that when the cells are cultured in 3D with OUR medium the toxicity of PTX is limited as it was not possible to derive the IC_50_ values for any of the cell types.

**FIGURE 7:**
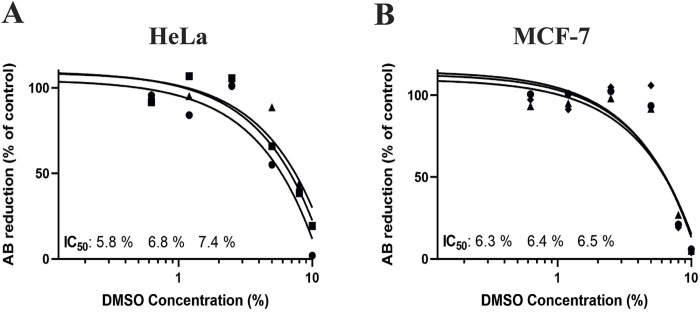
Dose-response curves for human cervical cancer HeLa cell **(A)** and human breast cancer MCF-7 cell 3D cultures treated with dimethyl sulfoxide (DMSO). The cells were seeded and incubated for 1 week in 3D cultures with OUR medium before the addition of DMSO to the concentrations 0.1, 0.6, 1.2, 2.5, 5, 8, and 10% as positive control of the possibility to induce cell death in 3D cultures. Control cells were treated with PBS. The toxicity was evaluated using the AlamarBlue™ assay and the results are expressed as the percentage reduction of AlamarBlue™ compared to control. The curves are drawn in GraphPad Prism 10 using all data from three independent experiments with n = 6 for each data point in the figure. The triangle, circle, and square symbols represent different experimental replicates.

Additionally, to confirm that it is possible to see toxicity in this 3D model system with OUR medium, we treated HeLa and MCF-7 cells with DMSO as a positive control. The 3D cultures of HeLa and MCF-7 were incubated in OUR medium for 1 week, followed by treatment with different concentrations of DMSO (0.1, 0.6, 1.2, 2.5, 5, 8, and 10%) for 72 h. Figure 7 presents the dose-response curves obtained from these experiments. The dose-response curves showed that DMSO exposure had a cytotoxic effect on cell proliferation of both MCF-7 and HeLa 3D cultures when they were grown in OUR medium (Figure 7). The average IC_50_ values obtained were 6.8 ± 0.8 and 6.4 ± 0.1 in HeLa and MCF-7 3D cultures, respectively. By demonstrating consistent IC_50_ values for DMSO in HeLa and MCF-7 3D cultures, our findings show the robustness of OUR medium as a cell growth medium for three human cell types and experimental conditions. Also, these findings highlight the significance of the potential cytotoxic effects of DMSO in 3D culture systems, particularly when utilized as positive control alongside screening other chemotherapeutic drugs. Altogether, these data present that OUR medium supports the growth of multiple cancer cell lines and allows for high-throughput compound screening.

### 3.6 Morphological evaluation of drug-treated cells grown in OUR medium

**FIGURE 8:**
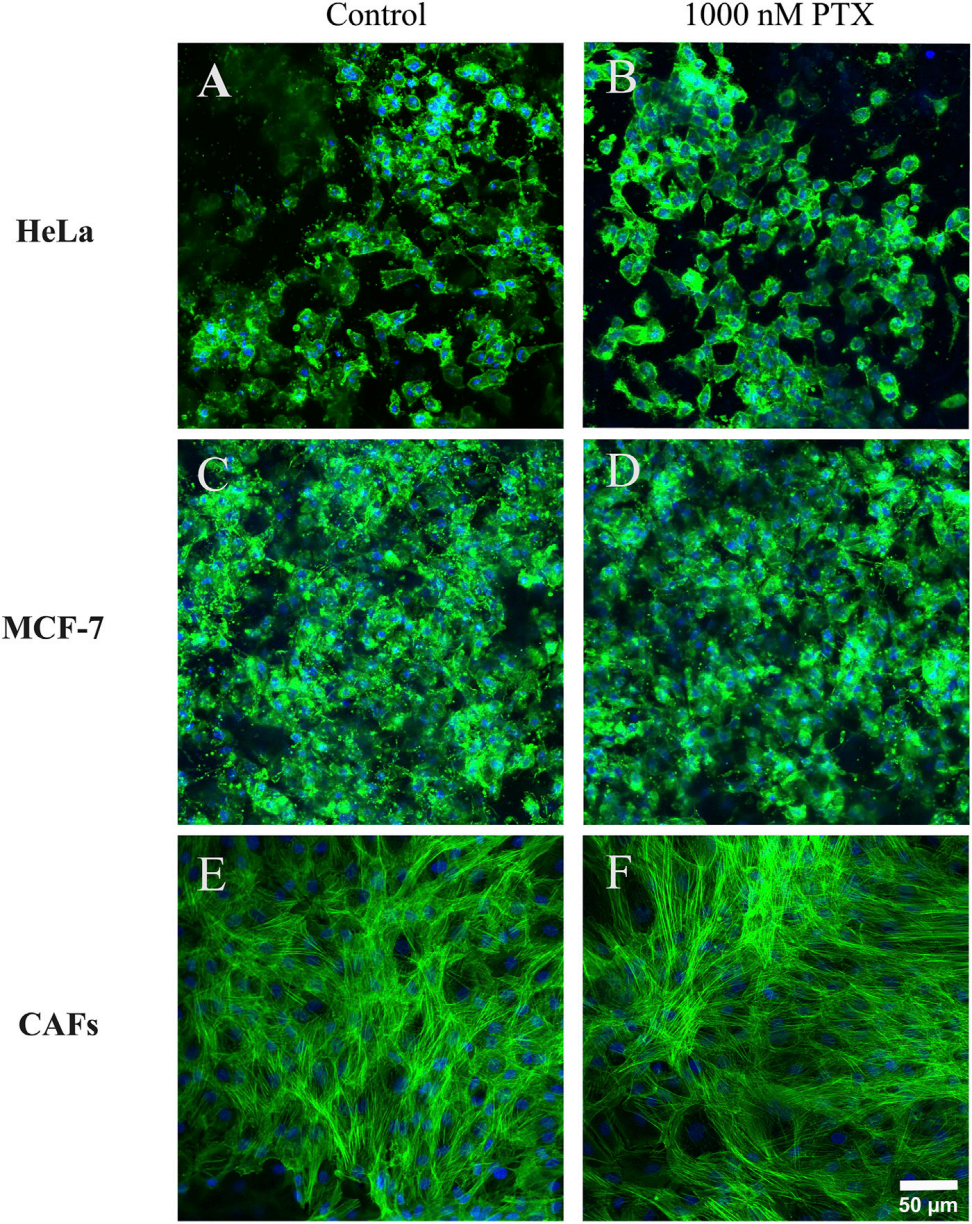
Confocal microscopy single plane images of control and paclitaxel (PTX)-treated 3D cultures of human cervical cancer HeLa cells **(A, B)**, human breast cancer MCF-7 cells **(C, D)**, and cancer-associated fibroblasts CAFs **(E, F)**, respectively. The images were taken on day 10 of incubation grown in OUR medium. PTX-treated cultures were exposed to 1000 nM PTX for 72 h. The images are representative of the cells treated with 1000 nM PTX in the dose-response data (Figure 6). The cultures were fixed in 4% formaldehyde and labeled to visualize actin filaments (green) and cell nuclei (blue). The confocal images are captured in the middle of the 3D cultures. The scale bar is 50 µm.

Confocal laser scanning microscopy was used to further study and validate the cellular response to PTX treatment using OUR medium in 3D cultures. Representative single confocal plane images of control cells treated with PBS with 0.1% DMSO and 1,000 nM PTX treated cells captured in the center of the fiber network of 3D cultures are shown in Figure 8. Figures 8A, C show the morphology of untreated HeLa and MCF-7 cancer cells is rounded while the untreated fibroblasts exhibited elongated and stretched-out morphology with a very clear actin cytoskeleton (Figure 8E). Comparing Figures 8A, B, the MCF-7 cells tend to form more tightly packed cellular aggregations than the HeLa cell in 3D cultures, which may be related to the slower growth rate and proliferation kinetics of the latter cells compared to MCF-7 cells (Table 1). With a slower proliferation rate, there may be fewer HeLa cells available to contribute to the formation of dense cellular aggregates compared to the more rapidly dividing MCF-7 cells. In the dense tumor-associated stroma *in vivo*, CAFs are usually organized in a distinct parallel-patterned and aligned arrangement, promoting various cellular behaviours such as cancer cell migration, communication, and tumor progression (Amatangelo et al., 2005). Here, we observe that untreated CAFs maintained an elongated, and spindle-shaped morphology in OUR medium (Figure 8E). Therefore, the cellular morphology of CAFs compared to untreated CAFs remains consistent and unchanged, when grown in OUR medium. Comparing Figures 8A, C, E, representing untreated, HeLa, MCF-7, and CAFs, respectively, with Figures 8B, D, F, which show PTX-treated HeLa, MCF-7, and CAFs, respectively, the minimal toxicity of PTX at 1,000 nM is apparent compared to the untreated cells in Figure 6. These findings further confirm the AlamarBlue results and suggest that OUR medium has the potential to support drug screening experiments. However, further studies with a full dose-response range are needed to fully validate its application in drug screening.

## 4 Discussion

The present study evaluated the suitability of OUR medium for cell adaptation, establishing a 3D culture system, and HTS 3D drug studies. The successful adaptation of HeLa cells, MCF-7 cells, and CAFs to the serum-free OUR medium aligns with a previous study that used gradual FBS reduction for transitioning cells to serum-free conditions (Rafnsdóttir et al., 2023). It has been suggested by van der Valk et al. (2010) that the cells should have a high viability of 90% or above during the adaptation steps when transitioning from an FBS-supplemented medium to a serum-free medium indicating robust growth and proliferation rate. As suggested by Ince et al. (2007), the use of Corning^®^ Primaria flasks played a key role in facilitating cell attachment and growth during the adaptation process. Corning^®^ Primaria flasks surfaces are both positively and negatively charged due to modification of nitrogen and oxygen-containing surface chemistry, respectively, compared to only negatively charged regular culture flasks. This modification facilitates cell attachment, spreading, and growth when cultured in OUR medium. It must be highlighted that the extracellular matrix in the tissue microenvironment contains proteins of highly negatively and positively charged due to the presence of various functional groups (Hynes, 2009; Lu et al., 2012). The observed morphological changes in HeLa and MCF-7 cells after adaptation to FBS-free OUR medium is consistent with previous studies. HeLa cells have shown some cytoplasmic retraction in other studies when they were grown in a serum-free medium supplemented with only human insulin, transferrin, hydrocortisone, epidermal growth factor, and fibroblast growth factor (Hutchings and Sato, 1978). Also, Abdeen et al. (2011) have reported alteration in HeLa cell morphology during the adaptation process from FBS-supplemented medium to Scharfenberg’s modification 6 (SMIF-6) protein-and serum-free medium where HeLa showed a more spindle-shaped morphology in serum-free medium. In contrast, CAFs did not exhibit significant changes in their cell area nor circularity index and therefore in their morphology when grown in OUR medium. This is in line with previous observations where human dermal fibroblasts and vesical fibroblasts showed no significant difference in morphology when cultured and maintained in bovine serum albumin-containing serum-free medium compared with an FBS-supplemented medium (Caneparo et al., 2022).

Time-lapse imaging provided valuable insights into the dynamic behavior and response of HeLa, MCF-7, and CAF cells when grown in OUR medium. The rapid cancer cell movements and active cell division observed in HeLa and MCF-7 cells further confirm that OUR medium supports cell division and therefore cell proliferation. On the other hand, it was observed that CAFs exhibited higher collective motility than cancer cells. Overall, the successful adaptation of HeLa, MCF-7, and CAF cells to OUR medium, as demonstrated through live imaging, contributes to our understanding of how OUR medium influences cellular dynamics and behavior over time.

In our laboratory, we have successfully adapted more than 23 different cell lines to grow and thrive in xeno-free OUR medium which can make it potentially a standard universal medium for cell culture. The results in this report indicate that MCF-7, HeLa, and CAFs adapted well to the OUR medium, maintaining comparable growth rates to those cultured in the FBS-supplemented medium. Also, The PDT obtained from the growth curves was compared with PDTs obtained in our laboratory, where the cells were cultured in a medium supplemented with FBS or donor herd horse serum. This suggests that the FBS-free OUR medium effectively supports the proliferation of these cell lines, providing a reliable alternative to the FBS-supplemented medium. Recent studies have reported 50.4 h and 72 h PDT within the same CAF sub-population derived from primary tumors (Pelon et al., 2020). In comparison, CAFs grown in OUR medium with a PDT of 46.3 h show a comparable proliferation rate with this report. Previously, Rafnsdóttir et al. (2023) have shown that the human pancreatic cancer cell line, MiaPaCa-2 cells, and mouse L929 cells maintained similar PDT when grown in OUR medium formulation as the FBS-supplemented medium. HeLa cells exhibited a longer PDT in OUR medium compared to the FBS-supplemented medium. Similarly, a longer PDT and slower growth rate were found for JIMT-1 cells in OUR medium compared to the FBS-supplemented medium (Rafnsdóttir et al., 2023). It is widely recognized that cell cycle length and PDT are inversely proportional and regulated by cell size and cell morphology (Cadart et al., 2018). Therefore, variations in PDT seem plausible when comparing *in vitro* to *in vivo* as we so far have not substantially compared cell behavior in the two conditions. Presumably, the most correct PDT would be the growth rate found in the human body where they are growing in their natural microenvironment. However, in the clinic, tumor doubling time, *e.g*., the time a tumor requires to double in volume, is commonly discussed rather than the cell’s PDT (Nakahashi et al., 2023).

3D cell culturing has been suggested to affect some aspects of cell behaviors, such as response to drug treatments, reflecting physiological conditions more accurately than traditional 2D cell culture (Jensen and Teng, 2020). The results from the 3D culturing experiments demonstrate that OUR medium can effectively support the expansion and infiltration of HeLa and MCF-7 cells into a 3D PCL-based fiber network. We have previously demonstrated that the electrospun PCL-based 3D model used in this study can provide suitable spacing and porosity, permitting cell infiltration into the scaffold’s electrospun network when they are grown in donor herd horse serum-supplemented medium (Malakpour-Permlid and Oredsson, 2021) and OUR medium (Rafnsdóttir et al., 2023). Therefore, the observed infiltration depths of 50 µm for HeLa cells and 62 µm for MCF-7 cells are comparable to previous results using donor herd horse serum-supplemented medium (Malakpour-Permlid and Oredsson, 2021). Additionally, this observation suggests that the absence of serum in OUR medium does not limit the expansion and ingrowth of cancer cells into the fiber network of our 3D model.

3D cell cultures are valuable platforms for HTS of anti-cancer drugs offering more reliable and efficient toxicity assessments (Wang and Jeon, 2022). This study shows the suitability of OUR medium for the 3D drug HTS application, demonstrating that HeLa, MCF-7, and CAFs grown in a 3D PCL fiber scaffold exhibit lower sensitivity to PTX compared to standard 2D systems. Further, the morphological analysis of PTX-treated cells in 3D cultures confirms the limited cytotoxicity of PTX at 1,000 nM in cells cultured in OUR medium. PTX, a well-established chemotherapeutic agent used currently for the treatment of cancer patients, disrupts microtubule dynamics during cell division which triggers pathways associated with cell death (Jordan and Wilson, 1998). Earlier, we have shown that OUR medium can be used for HTS dose-response testing of PTX in human JIMT-1 breast and MiaPaCa-2 pancreatic cancer cells cultured under a 3D setting (Rafnsdóttir et al., 2023). This observation aligns with the well-accepted concept that decreased drug responsiveness found in 3D cell culturing models is due to different intrinsic factors associated with the 3D microenvironment (Costard et al., 2021). Also, it has been reported that the reduced drug sensitivity of cells cultured in the electrospun 3D scaffold-based models can be in part due to the tortuous 3D fiber network structures, formation of cellular aggregations, and secreted ECM proteins within the 3D cultures which limit the distribution and availability of anti-cancer drugs to cells (Di Paolo and Bocci, 2007; Malakpour-Permlid, et al., 2024). Here, we did not aim to compare drug toxicity with cells grown in an FBS-supplemented medium, donor herd horse serum-supplemented medium, or within a 2D cell culture setting, as we have already investigated this in our previous research work. In our recently published study (Malakpour-Permlid, et al., 2024), we investigated the toxicity of PTX on HeLa and CAFs in the same 3D electrospun fiber scaffold, which we refer to as a non-homogenous 3D culture, using FBS-supplemented medium. The data demonstrated that similar to the results obtained when cells are grown in OUR medium, the PTX has limited toxicity on HeLa and CAFs in the FBS-supplemented medium, where it was not possible to derive the IC_50_ values in both cases (Malakpour-Permlid, et al., 2024). Also, we have previously used 3D HTS PCL fiber scaffolds to generate mini-tumors *in vitro* to assess the toxicity of PTX on JIMT-1 breast cancer cells and human normal fibroblasts using 5% heat-inactivated donor herd horse serum-supplemented medium (Malakpour-Permlid and Oredsson, 2021). Using the same 3D HTS PCL fiber scaffolds and OUR medium, we investigate the toxicity of PTX on JIMT-1 and MiaPaCa-2 cells (Rafnsdóttir et al., 2023). In our works, we have consistently found that the cells demonstrated significantly lower sensitivity to PTX when grown in 3D cultures compared to 2D cultures, which is in line with observations from other studies (Zhao et al., 2014; Baek et al., 2016). In this study, we use three new cell lines for 3D toxicity testing using OUR medium than in our previous published work. Thus, we expanded a wider range of cell lines that can be cultured in OUR medium and we demonstrated that combining OUR medium with 3D cultures presents a promising alternative to conventional 2D cultures for dose-response testing. Given our extensive collective information and data regarding different media, we chose to only culture the cells in 3D using OUR medium to further support and validate the notion that OUR medium is suitable for 3D drug screening. Previously, Zirvi and Hill (1988) demonstrated that treatment of human colon adenocarcinoma cells HT-29 with anti-cancer drugs, 5-fluorouracil and cisplatin, grown in both serum-free medium and 10% FBS-supplemented medium did not affect the results, and identical drug-response curves were derived. Consistent with these findings, it has been observed that cancer cells were found to show resistance to 5-fluorouracil efficacy when cultured in the presence of a physiologically relevant cell culture medium such as a human plasma-like medium (Cantor et al., 2017). Dubois et al. (2019) have used MDA-MB-231 and SUM1315 triple-negative breast cancer cells for the formation of 3D multicellular spheroids produced in a new synthetic serum-free medium for therapeutic drug screenings. The spheroid sensitivity to the anti-cancer chemotherapeutic drug epirubicin was evaluated in a serum-free medium and compared to the reference 10% FCS-supplemented medium. It was demonstrated that both MDA-MB-231 and SUM1315 spheroids showed lowered viability after treatment indicating similar cytotoxicity of anti-cancer drugs when grown in serum-free medium and FBS-supplemented medium (Dubois et al., 2019). The cytotoxicity effect of DMSO on cells cultured in OUR medium, used as a positive control, confirmed that OUR medium can be effectively used for cytotoxicity studies in 3D cultures. These findings are comparable with the studies performed using DMSO on cells grown in an FBS-supplemented medium. Previous reports have indicated that IC_50_ values of approximately 1.8% and 1.9% DMSO have been observed for MCF-7 and HeLa cancer cells cultured in 2D cultures with FBS-supplemented medium, respectively (Jamalzadeh et al., 2016; Demir et al., 2020). Our findings in the 3D cell culture show higher IC_50_ values with an average of 6.8 ± 0.8 and 6.4 ± 0.1 for HeLa and MCF-7 3D cultures, respectively. Also, it was demonstrated that treatment of pancreatic cancer cells with 10% DMSO led to total cell death in both monolayer 2D and miniaturized ECM-based 3D cell culture systems (Shelper et al., 2016). Thus, we believe that our data provides a strong indication of the reliability of using open access OUR medium across different experimental settings. Overall, our results demonstrate that the completely animal-component-free OUR medium may be a suitable growth medium for use in *in vitro* cytotoxicity studies for screening and selection of new therapeutic drugs.

## 5 Conclusion

In summary, the animal-component-free OUR medium formulation utilized here is a serum-free cell culture medium as an alternative to a widely-used FBS-supplemented medium that effectively supports the proliferation of cancer cells and normal cells and allows for HTS drug screening in 3D settings. A successful gradual adaptation procedure was implemented for the transition of cancer cells and CAFs grown in FBS-supplemented medium to OUR medium, resulting in the maintenance of their proliferation rate and morphology in OUR medium. For the first time, 3D cultures of human cervical cancer HeLa cells, human breast cancer MCF-7 cells or CAFs, were established using serum-free OUR medium. When growing the adapted cells in a 3D HTS PCL-based scaffold model, we found that the cells were able to distribute and infiltrate into the depths of the 3D PCL-based. Moreover, in a drug toxicity evaluation, we found that the toxicity of PTX was reduced in 3D culture using OUR medium which is in line with studies from our lab and other studies. Our animal-component-free system will offer a scientifically reproducible and efficient solution for drug screening applications in line with 3 R principles. We hope that our findings, as well as other studies using FBS-free media alternatives, promote the rapid removal of FBS from cell culture media, offering researchers an ethical, reliable, and consistent platform for conducting experiments in tumor biology and drug testing.

## Data Availability

The original contributions presented in the study are included in the article/Supplementary Material, further inquiries can be directed to the corresponding author.

## Abbreviations

2D, two-dimensional; 3D, three-dimensional; CAFs, cancer-associated fibroblasts; ECM, extracellular matrix; FBS, fetal bovine serum; HTS, high throughput screening; IC_50_, inhibitory concentration 50; OUR medium, Oredsson universal replacement medium; PDT, population doubling times; PTX, paclitaxel; PCL, polycaprolactone.

## References

[B1] Abdeen S. H. Abdeen A. M. El-Enshasy H. A. El Shereef A. A. (2011). HeLa-S3 cell growth conditions in serum-free medium and adaptability for proliferation in suspension culture. J. Biol. Sci. 11, 124–134. 10.3923/jbs.2011.124.134

[B2] Ackermann T. Tardito S. (2019). Cell culture medium formulation and its implications in cancer metabolism. Trends cancer 5, 329–332. 10.1016/j.trecan.2019.05.004 31208694 PMC6557711

[B3] Amatangelo M. D. Bassi D. E. Klein-Szanto A. J. Cukierman E. (2005). Stroma-derived three-dimensional matrices are necessary and sufficient to promote desmoplastic differentiation of normal fibroblasts. Ame. J. Pathol. 167, 475–488. 10.1016/S0002-9440(10)62991-4 PMC1603576 16049333

[B4] Asadian M. Chan K. V. Norouzi M. Grande S. Cools P. Morent R. (2020). Fabrication and plasma modification of nanofibrous tissue engineering scaffolds. Nanomater 10, 119. 10.3390/nano10010119 PMC7023287 31936372

[B5] Baek N. Seo O. W. Kim M. Hulme J. An S. S. (2016). Monitoring the effects of doxorubicin on 3D-spheroid tumor cells in real-time. OncoTargets Ther. 22, 7207–7218. 10.2147/OTT.S112566 PMC5125797 27920558

[B6] Balvers R. K. Kleijn A. Kloezeman J. J. French P. J. Kremer A. van den B. (2013). Serum-free culture success of glial tumors is related to specific molecular profiles and expression of extracellular matrix–associated gene modules. Neuro Oncol. 15, 1684–1695. 10.1093/neuonc/not116 24046260 PMC3829587

[B7] Barbosa M. A. Xavier C. P. Pereira R. F. Petrikaitė V. Vasconcelos M. H. (2022). 3D cell culture models as recapitulators of the tumor microenvironment for the screening of anti-cancer drugs. Cancers 14, 190. 10.3390/cancers14010190 PMC8749977 35008353

[B8] Cacciamali A. Villa R. Dotti S. (2022). 3D cell cultures: evolution of an ancient tool for new applications. Front. Physiol. 13, 836480. 10.3389/fphys.2022.836480 35936888 PMC9353320

[B9] Cadart C. Monnier S. Grilli J. Sáez P. J. Srivastava N. Attia R. (2018). Size control in mammalian cells involves modulation of both growth rate and cell cycle duration. Nat. Commun. 9, 3275. 10.1038/s41467-018-05393-0 30115907 PMC6095894

[B10] Caneparo C. Chabaud S. Fradette J. Bolduc S. (2022). Evaluation of a serum-free medium for human epithelial and stromal cell culture. Int. J. Mol. Sci. 23, 10035. 10.3390/ijms231710035 36077429 PMC9455993

[B11] Cantor J. R. Abu-Remaileh M. Kanarek N. Freinkman E. Gao X. Louissaint A. (2017). Physiologic medium rewires cellular metabolism and reveals uric acid as an endogenous inhibitor of UMP synthase. Cell. 169, 258–272.e17. 10.1016/j.cell.2017.03.023 28388410 PMC5421364

[B12] Chelladurai K. S. Christyraj J. D. S. Rajagopalan K. Yesudhason B. V. Venkatachalam S. Mohan M. (2021). Alternative to FBS in animal cell culture- an overview and future perspective. Heliyon 7, e07686. 10.1016/j.heliyon.2021.e07686 34401573 PMC8349753

[B13] Costard L. S. Hosn R. R. Ramanayake H. O'Brien F. J. Curtin C. M. (2021). Influences of the 3D microenvironment on cancer cell behaviour and treatment responsiveness: a recent update on lung, breast, and prostate cancer models. Acta Biomater. 132, 360–378. 10.1016/j.actbio.2021.01.023 33484910

[B14] Demir E. A. Demir S. Aliyazicioglu Y. (2020). *In vitro* cytotoxic effect of ethanol and dimethyl sulfoxide on various human cell lines. Kahramanmaraş Sütçü İmam Üniversitesi Tarım ve Doğa Derg. 23, 1119–1124. 10.18016/ksutarimdoga.vi.702702

[B15] Di Paolo A. Bocci G. (2007). Drug distribution in tumors: mechanisms, role in drug resistance, and methods for modification. Cur. Oncol. Rep. 9, 109–114. 10.1007/s11912-007-0006-3 17288875

[B16] Dubois C. Daumar P. Aubel C. Gauthier J. Vidalinc B. Mounetou E. (2019). The new synthetic serum-free medium OptiPASS promotes high proliferation and drug efficacy prediction on spheroids from MDA-MB-231 and SUM1315 triple-negative breast cancer cell lines. J. Clin. Med. 8, 397. 10.3390/jcm8030397 30901969 PMC6463163

[B17] Eiraku M. Watanabe K. Matsuo-Takasaki M. Kawada M. Yonemura S. Matsumura M. (2008). Self-organized formation of polarized cortical tissues from ESCs and its active manipulation by extrinsic signals. Cell. stem Cell. 3, 519–532. 10.1016/j.stem.2008.09.002 18983967

[B18] Gomez-Roman N. Stevenson K. Gilmour L. Hamilton G. Chalmers A. J. (2017). A novel 3D human glioblastoma cell culture system for modeling drug and radiation responses. Neuro Oncol. 19, 229–241. 10.1093/neuonc/now164 27576873 PMC5463789

[B19] Gottipamula S. Muttigi M. S. Kolkundkar U. Seetharam R. N. (2013). Serum-free media for the production of human mesenchymal stromal cells: a review. Cell. Prolif. 46, 608–627. 10.1111/cpr.12063 24118248 PMC6496935

[B20] Hutchings S. E. Sato G. H. (1978). Growth and maintenance of HeLa cells in serum-free medium supplemented with hormones. Proc. Nat. Acad. Sci. 75, 901–904. 10.1073/pnas.75.2.901 273251 PMC411365

[B21] Hynes R. O. (2009). The extracellular matrix: not just pretty fibrils. Sci. 326, 1216–1219. 10.1126/science.1176009 PMC3536535 19965464

[B22] Ince T. A. Richardson A. L. Bell G. W. Saitoh M. Godar S. Karnoub A. E. (2007). Transformation of different human breast epithelial cell types leads to distinct tumor phenotypes. Cancer Cell. 12, 160–170. 10.1016/j.ccr.2007.06.013 17692807

[B23] Jamalzadeh L. Ghafoori H. Sariri R. Rabuti H. Nasirzade J. Hasani H. (2016). Cytotoxic effects of some common organic solvents on MCF-7, RAW-264.7, and human umbilical vein endothelial cells. Avicenna J. Med. Biochem. 4, 10–33453. 10.17795/ajmb-33453

[B24] Jensen C. Teng Y. (2020). Is it time to start transitioning from 2D to 3D cell culture? Front. Mol. Biosci. 7, 33. 10.3389/fmolb.2020.00033 32211418 PMC7067892

[B25] Jochems C. E. Van Der Valk J. B. Stafleu F. R. Baumans V. (2002). The use of fetal bovine serum: ethical or scientific problem? Altern. Lab. Anim. 30, 219–227. 10.1177/026119290203000208 11971757

[B26] Jordan M. A. Wilson L. (1998). Microtubules and actin filaments: dynamic targets for cancer chemotherapy. Curr. Opin. Cell. Biol. 10, 123–130. 10.1016/S0955-0674(98)80095-1 9484604

[B27] Kojima Y. Acar A. Eaton E. N. Mellody K. T. Scheel C. Ben-Porath I. (2010). Autocrine TGF-β and stromal cell-derived factor-1 (SDF-1) signaling drives the evolution of tumor-promoting mammary stromal myofibroblasts. Proc. Natl. Acad. Sci. 107, 20009–20014. 10.1073/pnas.1013805107 21041659 PMC2993333

[B28] Larsson S. Rydén T. Holst U. Oredsson S. Johansson M. (2008). Estimating the total rate of DNA replication using branching processes. Bull. Math. Biol. 70, 2177–2194. 10.1007/s11538-008-9339-9 18818973

[B29] Liu Y. Hu T. Shen J. Li S. F. Lin J. W. Zheng X. H. (2006). Separation, cultivation and biological characteristics of oral carcinoma‐associated fibroblasts. Oral Dis. 12, 375–380. 10.1111/j.1601-0825.2005.01207.x 16792722

[B57] Louault K. Li R. R. DeClerck Y. A. (2020). Cancer-associated fibroblasts: understanding their heterogeneity. Cancers 12, 3108. 10.3390/cancers12113108 33114328 PMC7690906

[B30] Lu P. Weaver V. M. Werb Z. (2012). The extracellular matrix: a dynamic niche in cancer progression. J. Cell. Biol. 196, 395–406. 10.1083/jcb.201102147 22351925 PMC3283993

[B31] Malakpour-Permlid A. Oredsson S. (2021). A novel 3D polycaprolactone high-throughput system for evaluation of toxicity in normoxia and hypoxia. Toxicil. Rep. 8, 627–635. 10.1016/j.toxrep.2021.03.015 PMC8024882 33854950

[B32] Malakpour-Permlid A. Rodriguez M. M. Untracht G. R. Andersen P. E. Oredsson S. Boisen A. (2024). High-throughput non-homogenous 3D polycaprolactone scaffold for cancer cell and cancer-associated fibroblast mini-tumors to evaluate drug treatment response. Toxicol. Rep. 14, 101863. 10.1016/j.toxrep.2024.101863 39758801 PMC11699757

[B33] Nakahashi K. Nakatsuka M. Endo M. Shiono S. (2023). Tumor volume doubling time as a potential predictor of prognosis in clinical stage I lung squamous cell carcinoma. J. Thorac. Dis. 15, 3849–3859. 10.21037/jtd-23-292 37559608 PMC10407515

[B34] O'Brien J. Wilson I. Orton T. Pognan F. (2000). Investigation of the Alamar Blue (resazurin) fluorescent dye for the assessment of mammalian cell cytotoxicity. Eur. J. Biochem. 267, 5421–5426. 10.1046/j.1432-1327.2000.01606.x 10951200

[B35] Ored K. Li R. R. DeClerck Y. A. (2020). Cancer-associated fibroblasts: understanding their heterogeneity. Cancers 12, 3108. 10.3390/cancers12113108 33114328 PMC7690906

[B36] Oredsson S. Coecke S. van der Valk J. Vinken M. (2019). What is understood by “animal-free research”. Toxicol Vitro 57, 143–144. 10.1016/j.tiv.2019.03.001 30849472

[B37] Oredsson S. Malakpour-Permlid A. Weber T. Bajramovic J. (2024). A new animal-product-free, defined, and universal cell culture medium: easy to use, do-it-yourself, and beneficial for 2D and 3D culturing of normal and cancer cells. 10.13140/RG.2.2.27131.53284

[B38] Pamarthy S. Sabaawy H. E. (2021). Patient derived organoids in prostate cancer: improving therapeutic efficacy in precision medicine. Mol. Cancer 20, 125. 10.1186/s12943-021-01426-3 34587953 PMC8480086

[B39] Pelon F. Bourachot B. Kieffer Y. Magagna I. Mermet-Meillon F. Bonnet I. (2020). Cancer-associated fibroblast heterogeneity in axillary lymph nodes drives metastases in breast cancer through complementary mechanisms. Nat. Commun. 11, 404. 10.1038/s41467-019-14134-w 31964880 PMC6972713

[B40] Pilgrim C. R. McCahill K. A. Rops J. G. Dufour J. M. Russell K. A. Koch T. G. (2022). A review of fetal bovine serum in the culture of mesenchymal stromal cells and potential alternatives for veterinary medicine. Front. Veter. Sci. 9, 859025. 10.3389/fvets.2022.859025 PMC9111178 35591873

[B41] Rafnsdóttir Ó. B. Kiuru A. Tebäck M. Friberg N. Revstedt P. Zhu J. (2023). A new animal product free defined medium for 2D and 3D culturing of normal and cancer cells to study cell proliferation and migration as well as dose response to chemical treatment Toxicology reports *Toxicol. Rep* . Toxicol. Rep. 10, 509–520. 10.1016/j.toxrep.2023.04.001 37396848 PMC10313884

[B42] Recek N. Resnik M. Motaln H. Lah-Turnšek T. Augustine R. Kalarikkal N. (2016). Cell adhesion on polycaprolactone modified by plasma treatment. Int. J. Polym. Sci. 2016, 1–9. 10.1155/2016/7354396

[B43] Sakthiselvan P. Meenambiga S. S. Madhumathi R. (2019). Kinetic studies on cell growth. Cell. growth 13. 10.5772/intechopen.84353

[B44] Shelper T. B. Lovitt C. J. Avery V. M. (2016). Assessing drug efficacy in a miniaturized pancreatic cancer *in vitro* 3D cell culture model. Assay. Drug Dev. Technol. 14, 367–380. 10.1089/adt.2016.737 27552143

[B45] Silva T. M. Cirenajwis H. Wallace H. M. Oredsson S. Persson L. (2015). A role for antizyme inhibitor in cell proliferation. Amino Acids 47, 1341–1352. 10.1007/s00726-015-1957-6 25813938 PMC4458265

[B46] Sinacore M. S. Drapeau D. Adamson S. R. (2000). Adaptation of mammalian cells to growth in serum-free media. Mol. Biotechnol. 15, 249–257. 10.1385/MB:15:3:249 10986701

[B47] Tang L. (2019). Investigating heterogeneity in HeLa cells. Nat. Methods 16, 281. 10.1038/s41592-019-0375-1 30923376

[B48] Tsuji K. Ojima M. Otabe K. Horie M. Koga H. Sekiya I. (2017). Effects of different cell-detaching methods on the viability and cell surface antigen expression of synovial mesenchymal stem cells. Cell. Transpl. 26, 1089–1102. 10.3727/096368917X694831 PMC5657749 28139195

[B49] Van der Valk J. (2022). Fetal bovine serum—a cell culture dilemma. Sci. 375, 143–144. 10.1126/science.abm1317 35025663

[B50] Van der Valk J. Bieback K. Buta C. Cochrane B. Dirks W. G. Fu J. (2018). Fetal bovine serum (FBS): past–present–future. ALTEX-Alter. Anim. Ex. 35, 99–118. 10.14573/altex.1705101 28800376

[B51] Van der Valk J. Brunner D. De Smet K. Svenningsen Å. F. Honegger P. Knudsen L. E. (2010). Optimization of chemically defined cell culture media–replacing fetal bovine serum in mammalian *in vitro* methods. Toxicol. vitro 24, 1053–1063. 10.1016/j.tiv.2010.03.016 20362047

[B52] Wang Y. Jeon H. (2022). 3D cell cultures toward quantitative high-throughput drug screening. Trends Pharmacol. Sci. 43, 569–581. 10.1016/j.tips.2022.03.014 35504760

[B53] Weber T. Wiest J. Oredsson S. Bieback K. (2022). Case studies exemplifying the transition to animal component-free cell culture. Altern. Lab. Anim. 50, 330–338. 10.1177/02611929221117999 35983799

[B54] Zasadil L. M. Andersen K. A. Yeum D. Rocque G. B. Wilke L. G. Tevaarwerk A. J. (2014). Cytotoxicity of paclitaxel in breast cancer is due to chromosome missegregation on multipolar spindles. Sci. Transl. Med. 6, 229ra43. 10.1126/scitranslmed.3007965 PMC4176609 24670687

[B55] Zhao Y. Yao R. Ouyang L. Ding H. Zhang T. Zhang K. (2014). Three-dimensional printing of Hela cells for cervical tumor model *in vitro* . Biofabrication 6, 035001. 10.1088/1758-5082/6/3/035001 24722236

[B56] Zirvi K. A. Hill G. J. (1988). Comparison of growth and drug response of human tumor cells in serum‐free and serum‐supplemented media in human tumor‐clonogenic assay. J. Surg. Oncol. 38, 88–93. 10.1002/jso.2930380206 3379971

